# Do Images of Biskyrmions Show Type‐II Bubbles?

**DOI:** 10.1002/adma.201806598

**Published:** 2019-03-07

**Authors:** James C. Loudon, Alison C. Twitchett‐Harrison, David Cortés‐Ortuño, Max T. Birch, Luke A. Turnbull, Aleš Štefančič, Feodor Y. Ogrin, Erick O. Burgos‐Parra, Nicholas Bukin, Angus Laurenson, Horia Popescu, Marijan Beg, Ondrej Hovorka, Hans Fangohr, Paul A. Midgley, Geetha Balakrishnan, Peter D. Hatton

**Affiliations:** ^1^ Department of Materials Science and Metallurgy University of Cambridge 27 Charles Babbage Road Cambridge CB3 0FS UK; ^2^ Faculty of Engineering and Physical Sciences University of Southampton Southampton SO17 1BJ UK; ^3^ Department of Physics University of Durham Durham DH1 3LE UK; ^4^ Department of Physics University of Warwick Coventry CV4 7AL UK; ^5^ School of Physics and Astronomy University of Exeter Exeter EX4 4QL UK; ^6^ Synchrotron SOLEIL Saint Aubin, BP 48 91192 Gif‐sur‐Yvette France; ^7^ European XFEL GmbH Holzkoppel 4 22869 Schenefeld Germany

**Keywords:** biskyrmions, Lorentz transmission electron microscopy, magnetic bubbles, skyrmions, X‐ray holography

## Abstract

The intense research effort investigating magnetic skyrmions and their applications for spintronics has yielded reports of more exotic objects including the biskyrmion, which consists of a bound pair of counter‐rotating vortices of magnetization. Biskyrmions have been identified only from transmission electron microscopy images and have not been observed by other techniques, nor seen in simulations carried out under realistic conditions. Here, quantitative Lorentz transmission electron microscopy, X‐ray holography, and micromagnetic simulations are combined to search for biskyrmions in MnNiGa, a material in which they have been reported. Only type‐I and type‐II magnetic bubbles are found and images purported to show biskyrmions can be explained as type‐II bubbles viewed at an angle to their axes. It is not the magnetization but the magnetic flux density resulting from this object that forms the counter‐rotating vortices.

## Graphical abstract

**Research on skyrmions** has yielded reports of biskyrmions: bound pairs of counter‐rotating vortices of magnetization. This study combines electron microscopy, X‐ray holography, and simulations to investigate biskyrmions in MnNiGa. It demonstrates that images of biskyrmions can be explained as type‐II magnetic bubbles. It is not the magnetization but the magnetic flux density from this object that forms the counter‐rotating vortices.

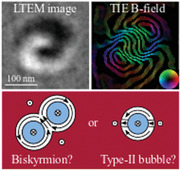

A magnetic skyrmion is a localized magnetic configuration with an integer, nonzero topological charge which can occur in magnetic materials.^1^ The Bloch skyrmions considered here resemble magnetic vortices but have an integer topological charge rather than $\pm \frac{1}{2}$. The concept of a skyrmion was introduced in 1961 in the context of nuclear physics^2^ and in 1989, magnetic skyrmions were predicted^3^ to occur as a result of the competition between the Heisenberg exchange energy and the Dzyaloshinskii–Moriya interaction.^4^ We use the term “DM‐skyrmions” to refer to such objects.

DM‐skyrmions were found experimentally^5^ in bulk MnSi in 2009. This prompted the recent intense research effort as magnetic skyrmions can be moved by electrical currents a million times smaller than those required to move ferromagnetic domain walls, making them promising objects for spintronic applications, notably racetrack computer memories.^6^, ^7^, ^8^ The Dzyaloshinskii–Moriya interaction and hence DM‐skyrmions can occur only in magnetic systems which lack an inversion symmetry either due to interfaces between different materials^9^, ^10^ or because the crystal is non‐centrosymmetric.^5^, ^11^, ^12^

In contrast, magnetic bubbles can occur in centrosymmetric magnets and despite resembling DM‐skyrmions, their origin is different.^13^, ^14^ Magnetic bubbles can be generated in thin sheets of magnetic material where the easy axis is oriented out‐of‐plane. If the uniaxial magnetocrystalline anisotropy is sufficiently large, the magnetization points out‐of‐plane and striped magnetic domains form as shown by the micromagnetic simulation in the left‐hand panel of **Figure** **1**a.

**Figure 1:**
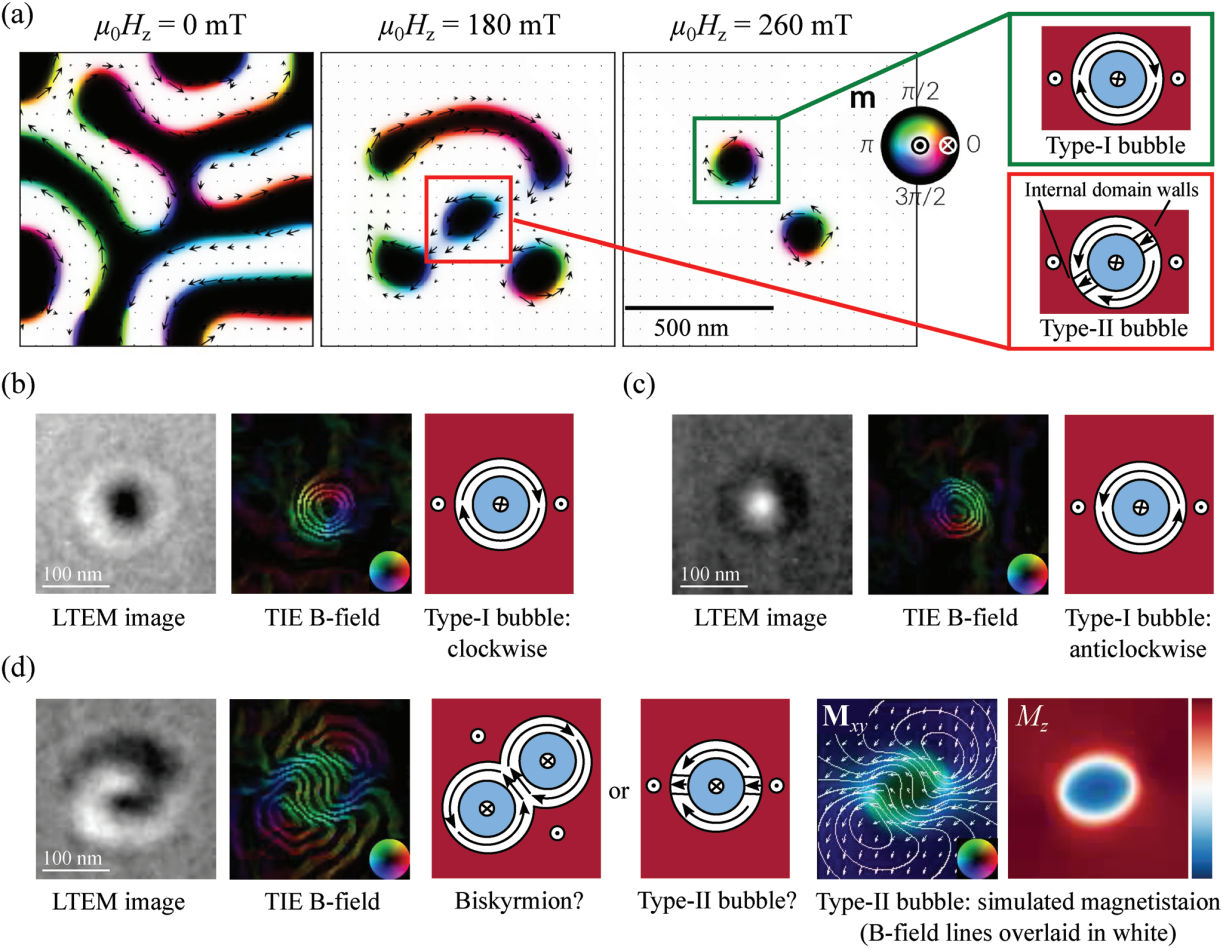
a) Micromagnetic simulations showing the formation of different types of magnetic bubble as the applied field *H*_*z*_ is increased. b–d) Electron microscopy images showing bubbles in MnNiGa together with the projected B‐field reconstructed from a defocus series of such images and the magnetic states to which they correspond with arrows indicating the magnetization direction. Microscopy images like in (d) have been identified as biskyrmions but more likely show type‐II bubbles (see text for details). The images were acquired at room temperature in an out‐of‐plane applied field of 233 mT at defocus Δ*f* = −1.41 mm. The projected B‐field lines correspond to the cosine of 100 times the phase of the exit wavefunction of the electron beam and their direction is indicated by the inset color wheels. The right‐hand panel of (d) labeled **M**_*xy*_ shows a micromagnetic simulation of the projected magnetization normal to the electron beam of a type‐II bubble viewed at 9.5° to its axis, indicated by arrows and colors. B‐field lines are overlaid in white and closely resemble the TIE reconstruction. The final panel shows the projected magnetization component parallel to the beam, *M*_*z*_, the strength indicated by the red‐blue color bar with red denoting positive values, blue negative, and white zero.

When a magnetic field is applied out‐of‐plane (*H*_*z*_), domains with an antiparallel magnetization shrink until they break into roughly circular domains called magnetic bubbles as shown in Figure 1a (and in more detail in Video S1 and Figure S2, Supporting Information). Different sizes and densities of magnetic bubbles result from different field treatments and repeated field pulses can be used to create a dense hexagonal array of bubbles.^13^ Magnetic bubbles of size 0.5 mm were likely first imaged in 1959 in oxides such as YFeO_3_ using polarized light microscopy^15^ and designs for a computer memory using bubbles a few micrometers in diameter were given in a 1967 review.^16^ Much smaller bubbles around 100 nm in diameter have been produced in other materials as part of the recent research into magnetic skyrmions.^17^, ^18^, ^19^, ^20^

Two types of bubbles can be distinguished in Figure 1a and are sketched in the right‐hand panel. In a type‐I bubble,^21^ the magnetization in the domain wall surrounding the bubble's core circulates either clockwise or anticlockwise with equal probability. This is different from skyrmions where the magnetic chirality is determined by the chirality of the crystal in bulk systems and the interfacial symmetry breaking in thin films. Like skyrmions, type‐I bubbles have a topological charge of 1 and so are topologically nontrivial and are sometimes called “skyrmion‐” or “skyrmionic‐bubbles”.^22^, ^23^, ^24^, ^25^ In a type‐II bubble (also called the “onion state”—the name derives from a related structure in magnetic rings^23^, ^26^), the circulation sense of the magnetization reverses at points we term “internal domain walls.” Our simulations show they have topological charge 0 in agreement with ref. 23 making these magnetic structures topologically trivial.

The left‐hand panels of Figures 1b,c show electron microscopy images of type‐I bubbles in Mn_0.325_Ni_0.324_Ga_0.350_ (subsequently referred to as MnNiGa) and it can be seen that they appear as a black circle for one circulation direction and white for the other at a given defocus. Electron microscopy images are sensitive to the component of the magnetic flux density normal to the electron beam averaged through the sample thickness along the beam direction—a quantity we term the “projected B‐field.” The projected B‐field can be recovered from a series of such images acquired at different defoci using the transport of intensity equation (TIE)^27^ as shown in the central panels of (b) and (c). In a type‐I bubble there is no stray field so the magnetization is proportional to the B‐field and the bubble type and circulation sense are readily identified as shown in the right‐hand panels.

In 2014, images with both black and white features like that shown in the left panel of Figure 1d were reported in La_2−2*x*_Sr_1+2*x*_Mn_2_O_7_ (*x* = 0.315)^18^ and the same state has been identified in (Mn_1−*x*_Ni_*x*_)_0.65_Ga_0.35_ (*x* = 0.5)^20^ and amorphous Fe–Gd thin films with perpendicular magnetic anisotropy,^19^ none of which exhibit the Dzyaloshinskii–Moriya interaction. These images were identified as showing biskyrmions because the projected B‐field reconstructed from such images showed two counter rotating vortices as seen in Figure 1d.

No mathematical model has been proposed for a biskyrmion but it consists of two cores (regions where the magnetization opposes the applied field) shown in blue in the sketch in Figure 1d, surrounded by counter‐rotating vortices. Images identified as showing biskyrmions range in appearance from black and white semicircles to the interlocking black‐white contrast of Figure 1d sometimes called “Yin‐Yang.”^23^ Yin‐Yang contrast can be seen in images from La_1.37_Sr_1.63_Mn_2_O_7_ (Figure 2c of ref. 18) and MnNiGa (Figures 2a and 3 of ref. 28).

Here, we acquired images of magnetic bubbles from MnNiGa using X‐ray holography with extended references^29^, ^30^ and Lorentz transmission electron microscopy and compared these with simulations. The right two panels of Figure 1d show a micromagnetic simulation of a type‐II bubble when viewed at an angle to its axis with the projected B‐field shown by white lines. Even though the simulated bubble has a single core as seen in *M*_*z*_, the B‐field has two counter‐rotating vortices and closely resembles the B‐field derived from experimental images. Thus we show that the counter‐rotating vortices in the B‐field need not correspond to similar vortices in the magnetization and the object identified as a biskyrmion is more likely a conventional type‐II bubble viewed at an angle to its axis.

Only a few studies have reported generating biskyrmions in computer simulations such as refs. 31 and 32 which are based on a Heisenberg‐like spin model. The former paper deals only with thin samples using reduced parameters whereas the latter considers frustrated exchange interactions. We chose to use micromagnetic simulations as they are best suited to the length scales relevant to our experiments. We could not find a biskyrmion state by varying the material parameters within the range estimated from experiments, irrespective of whether there was a Dzyaloshinskii–Moriya interaction, and observed only type‐I and type‐II bubbles.

To search for biskyrmions experimentally, we acquired X‐ray holograms which give the out‐of‐plane component of the magnetization averaged through the sample thickness (Section S3, Supporting Information). The MnNiGa sample used was a 200 nm thick, 10 × 5 µm single crystal plate with its large surfaces normal to the [001] magnetic easy axis. It was cut from a single grain of a polycrystal using a focused ion‐beam microscope and the sample used for transmission electron microscopy was cut from the same grain (Sections S2– S4, Supporting Information). MnNiGa has a hexagonal crystal structure with space group *P*6_3_/*mmc* and lattice parameters *a* = *b* = 4.15 Å and *c* = 5.33 Å (Section S1, Supporting Information).

The sample was viewed in [001] and a field sweep from 0 to 284 mT was first carried out at room temperature with the field applied normal to the sample's surface. At low field, stripe domains were observed. As the field was increased, those domains opposed to the field first narrowed and then fragmented above 250 mT to become a sparse array of single‐cored bubbles each with an average diameter of 120 nm separated by 650 nm. This process of bubble formation closely resembled the micromagnetic simulations in Figure 1.

The sample was then heated above its Curie temperature of 350 K and cooled back to room temperature in a field of 35 mT which produced a dense array of bubbles. The field was then swept from 0 to 400 mT and **Figure** **2**a shows images acquired during this procedure. It can be seen from Figures 2a,b that the bubbles shrank to about half their original size as the field was increased although their spacing remained constant to within the margin of error. At no point was there any indication of magnetic features with the double core that would be expected from a biskyrmion.

**Figure 2:**
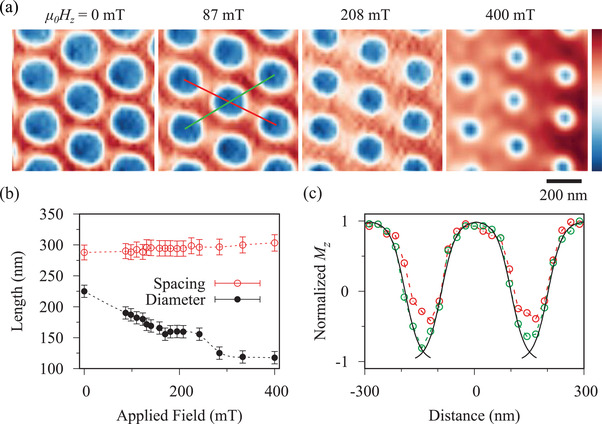
a) X‐ray holograms showing the component of the magnetization normal to the specimen (*M*_*z*_) at room temperature as the field applied normal to the specimen plane is increased. The strength of *M*_*z*_ is indicated by the color bar with red denoting positive values, blue negative, and white zero. b) The effect of increasing the applied field on the average center‐to‐center spacing of the bubbles and their diameter. c) Linescans taken in the directions shown in (a) by the red and green lines. Solid black lines indicate hyperbolic tangent fits to the magnetic domain walls (see text).

It is difficult to distinguish type‐I and II bubbles from an X‐ray hologram as the out‐of‐plane magnetization is very similar in both cases but the linescans in Figure 2c indicate that these are likely to be type‐II bubbles. Scanning in one direction, the magnetization reverses between the bubbles and can be fit with the expected hyperbolic tangent profile^33^ with a domain wall width of 47 ± 5 nm. Scanning in the other direction, the magnetization does not fully reverse as indicated by the white haze between the bubbles in Figure 2a implying an in‐plane component of the magnetization persists between the bubbles. The micromagnetic simulation in Figure 1a for 180 mT shows this happens near the internal domain walls of closely spaced type‐II bubbles.

To obtain experimental information on the in‐plane component of the magnetization, Lorentz transmission electron microscopy images were acquired. Such images are not sensitive to the magnetization itself but to the projected B‐field. The sample was a single crystal MnNiGa lamella thinned on the (001) plane to two different thicknesses: 110–180 nm in the thinner region and 200–230 nm in the thicker (Section S6 and Figure S5, Supporting Information).

Again, the process by which bubbles formed closely resembled the predictions of the micromagnetic simulations shown in Figure 1a. Only striped domains were observed at room temperature when an initial field of 143 mT was applied normal to the lamella. When the field was increased to 233 mT, coexisting bubbles and striped domains were observed as shown in **Figure** **3**a, taken from the thicker region of the sample. All the electron microscopy images herein have the same orientation indicated by the crystallographic directions in square brackets. The black‐white contrast indicated the bubbles were the same object previously identified as a biskyrmion. Figure S5 in the Supporting Information shows that the same state also occurred in the thinner region even though the diameter of the bubbles was 40% less and their spacing 30% less than those in the thicker region.

**Figure 3:**
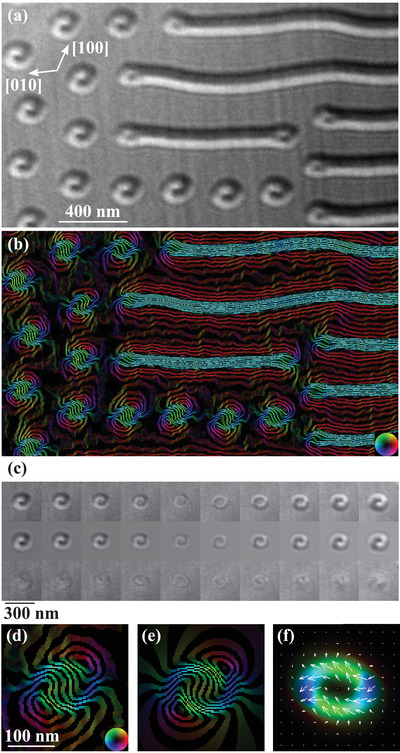
a) Electron microscopy image showing striped domains and bubbles in MnNiGa at room temperature in an out‐of‐plane applied field of 233 mT with defocus Δ*f* = −1.410 mm. b) Projected B‐field calculated from a defocus series of such images. The color wheel shows the direction of the field and the field lines correspond to the cosine of 100 times the phase of the exit wavefunction of the electron beam. c) Top row: experimental defocus series of one bubble taken at defoci Δ*f* = −1.410, −1.128, −0.846, −0.564, −0.282, 0.282, 0.564, 0.846, 1.128, and 1.410 mm (left to right). Middle row: simulated defocus series. Bottom row: difference between experimental and simulated images. d) Projected B‐field for this bubble calculated from the defocus series and e) from the simulated bubble. f) Simulated projected magnetization from which (e) was calculated.

Ten such images were acquired under the same conditions, each with a different defocus and the transport of intensity equation^27^ was used to calculate the projected B‐field, resulting in Figure 3b. It can be seen that each bubble has the two counter‐rotating vortices characteristic of a “biskyrmion.” As the sample thickness and defocus had been measured (Section S4, Supporting Information), the B‐field was obtained quantitatively and in absolute units.

The projected B‐field was used as an inspiration to create an analytic model for the magnetization. The model is described in Section S7 in the Supporting Information and represents the magnetization averaged through the thickness of the sample in the electron beam direction. It consists of a modified type‐II bubble in which the component of the magnetization normal to the electron beam does not follow the domain wall surrounding the bubble but is inclined to it as shown in Figure 3f. The likelihood of such a bubble occurring is discussed later but for now it serves to show that the data can be explained by a bubble with a single core without the need to invoke a biskyrmion.

Defocused images were simulated using this model as described in the Section S7 in the Supporting Information. The parameters of the model such as the saturation magnetization, domain wall width, and angle at which the magnetization was inclined to the wall were varied using a simplex algorithm^34^ to minimize the normalized mean square difference between the experimental and simulated images (χ^2^). Figure 3c shows the results of this for one of the bubbles.

Usually χ^2^ close to 1 indicates a good fit but since the images showed additional contrast from slight thickness undulations due to ion milling, regions showing no magnetic contrast were used to establish that χ^2^ between 2 and 3 indicated a good fit. Our fit gave χ^2^ = 6.3 and it can be seen from the difference images in Figure 3c that the fit is close but not perfect. It nevertheless reproduces all the features of the images.

The projected B‐field calculated from the defocus series using the transport of intensity equation is shown in Figure 3d and it can be seen that it closely resembles the B‐field calculated from the analytic model of the magnetization shown in (e). The magnetization itself is shown in (f) and it can be seen that it has a single core rather than the double core required for a biskyrmion.

The fitting procedure was repeated for 19 bubbles in the same defocus series yielding a saturation magnetization μ_0_ *M*_*s*_ = 0.0551 ± 0.0006 T and a domain wall width of δ = 47 ± 1 nm which is in good agreement with the value of 47 ± 5 nm derived from X‐ray holography. The average values of the other parameters used in the model are listed in Table S1 in the Supporting Information.

We also found that different types of bubbles can coexist and transitions between them can be stimulated by abruptly tilting the sample by 1° in an out‐of‐plane field of 233 mT. We acquired videos of these transitions and found that after tilting, most bubbles retained their original state but a few changed their state for up to a minute after tilting with each bubble changing faster than the frame rate of 1.15 s. The different transitions are shown in **Figure** **4**. We observed type‐I bubbles reversing their helicity (Figure 4a,b); type‐I bubbles of either helicity transforming into bubbles exhibiting “biskyrmion” contrast ((c) and (d)) and vice‐versa ((e) and (f)) as well as type‐I bubbles and “biskyrmions” transforming into the spin‐aligned state and so vanishing from the image ((g), (h), and (i)).

**Figure 4:**
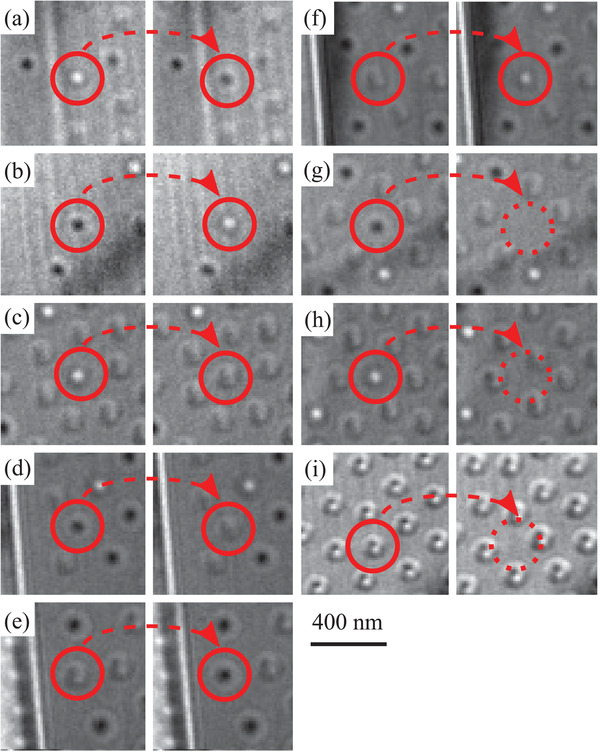
a–i) Transformations of bubbles in MnNiGa (see text). The transitions occurred after a sudden tilt of 1° in an out‐of‐plane field of 233 mT and the right‐hand images were acquired 1.15 s after the left‐hand ones at Δ*f* = 1.682 mm as part of a video. Unlike the other images, those in (i) were acquired incidentally as part of a defocus series. The two images were taken 15 s apart with the left at Δ*f* = 0.846 and the right at Δ*f* = 1.128 mm.

As we argue, the “biskyrmions” are most likely type‐II bubbles. Yu et al.^17^ have shown that transitions between type‐I and type‐II bubbles occur this way in their electron microscopy study of magnetic bubbles in BaFe_12−*x*−0.05_Sc_*x*_Mg_0.05_O_19_, *x* = 0.16. They found that transitions between type‐I and II bubbles can be stimulated by tilting the sample 1.5° in a vertical field of 80 mT. Like us, they also found that bubbles form by the fragmentation of stripe domains and that bubbles of both type and helicity can coexist.

We have shown that images purported to show biskyrmions can be explained by the analytic model described in Section S7 in the Supporting Information in which a type‐II bubble with a single core is modified so that the magnetization does not follow the domain wall surrounding the bubble but is inclined to it. We now discuss whether such a magnetic structure is plausible.

To make this assessment, we performed micromagnetic simulations described in Section S5 in the Supporting Information. First we randomly initialized the magnetic configuration and performed the field sweep shown in Figure 1a; Video S1, Supporting Information. This produced only conventional type‐I and type‐II bubbles. We then seeded the simulation with a type‐II bubble and the structure relaxed to give a conventional type‐II bubble. When seeded with the modified type‐II structure, the structure proved unstable and turned into the saturated state. Thus it is likely that the modified type‐II bubble does not represent the true structure of the bubble but is the result of averaging the 3D structure through the sample thickness.

The 3D structure of a type‐II bubble produced by micromagnetic simulations is shown in **Figure** **5**a and it can be seen that it varies considerably throughout the thickness of the specimen. The wall surrounding the bubble is type‐II near the center but near the surfaces the magnetization points radially. This was observed in simulations of samples from 80 to 200 nm thickness (Figure S4, Supporting Information) and is a well‐known effect caused by the magnetization near the surface aligning with the stray field.^35^, ^36^, ^37^ It was predicted in 1973 and soon could be confirmed experimentally using the new techniques of X‐ray and electron magnetic tomography which are being developed to map magnetic structures in three dimensions.^38^, ^39^

**Figure 5:**
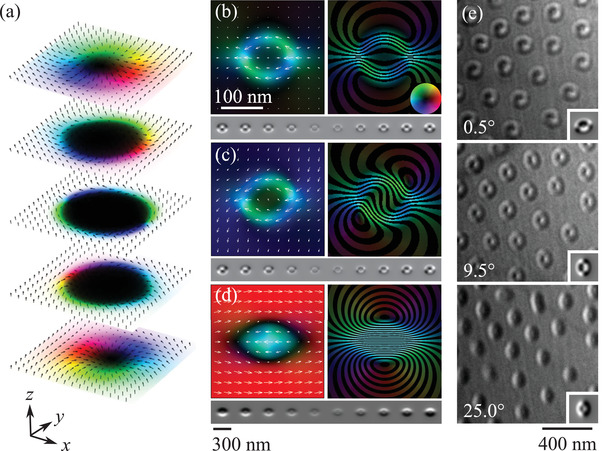
a) Magnetization of a simulated type‐II bubble displayed in three dimensions as equally spaced slices. The sample surfaces lie in the *xy* plane and the line joining the internal domain walls is parallel to *x*. The uniaxial magnetocrystalline anisotropy and applied field are parallel to *z*. b) Projected magnetization **M**_*xy*_ (left) and B‐field (right) for the electron beam parallel to the bubble's axis *z* with a defocus series for these conditions shown beneath. c) Projected magnetization and B‐field for a sample tilted 9.0° about *x* and 3.5° about *y* with respect to the electron beam. The same simulation is shown in Figure 1d. The associated defocus series is shown beneath. d) Projected magnetization and B‐field for a sample tilted 25° about *y*. Its defocus series is shown beneath. The simulated defocus series have the same defoci as those in Figure 3. e) Electron microscopy images of magnetic bubbles acquired with an out‐of‐plane applied field of 201 mT with defocus Δ*f* = 0.872 mm at room temperature. Each image shows the same array of bubbles as the specimen is tilted about a horizontal axis by the angles given in the bottom left. Inserts at the bottom right show simulated images for tilt angles −9°, 0°, and 16°.

If the bubble's magnetization is averaged through the specimen thickness along its axis, the contributions above and below the central plane cancel and the bubble appears as a conventional type‐II bubble as shown in Figure 5b with the corresponding defocus series shown below. There is no guarantee, however, that the electron beam will be parallel to the bubble's axis and so we investigated the appearance of such a structure if projected at an angle to its axis.

It proved difficult to recreate the same size of bubble as that seen experimentally as the bubble's size depends on the interaction with neighboring bubbles, the sample edges, and pinning sites. Experimentally, we found that the magnetic states that occurred were history dependent. For example, the bubbles in Figure 3 had an average diameter of 117 nm. After the sample was tilted to 32° and back to 0° maintaining a field of 233 mT parallel to the electron beam, the average diameter was 75 nm.

We could simulate isolated bubbles of diameter 120 nm, close to the observed diameter of 117 nm, in 100 nm thick samples. Simulations close to the experimental thickness of 200 nm produced bubbles at least double the radius although they had the same structure (Figure S4, Supporting Information). As the appearance of the images depends crucially on the size of the bubble, we used the simulations for 100 nm thickness and renormalized the thickness and saturation magnetization to match the experimental values to calculate the appearance of the electron microscopy images. The tilting angles we quote here are scaled to match a 200 nm thickness.

The effect of tilting the specimen 9.0° about *x* and 3.5° about *y* is simulated in Figure 5c and it can be seen that the projected B‐field closely resembles that derived from experimental measurements shown in Figure 3 as does the simulated defocus series below. A higher tilt angle of 25° with respect to *y* (Figure 5d) produced the half‐white half‐black appearance of the images identified as biskymrions in refs. 18, 19, 20, 40, 41, 42.

To confirm these predictions, we acquired images of the magnetic bubbles shown in Figure 5e and it can be seen that their appearance changed profoundly with the tilt angle. When the specimen plane was nearly normal to the electron beam (0.5°), the bubbles had the Yin‐Yang appearance discussed earlier. At 9.5°, the images resembled the simulations with the electron beam parallel to the axis of the bubble. At 25.0°, the images appeared half‐white half‐black.

Simulations of these images are shown as insets and it can be seen that there is a close resemblance. The relative tilt angles between the simulated images are in good agreement with those measured experimentally. There is a 9° offset in the absolute angle which may be because the sample's surface is not quite normal to the easy axis as would be typical for this type of specimen preparation. We estimated this offset to be around 7° from the tilt required to reach the [001] easy axis from the specimen's initial position in the microscope. Given the hysteresis in the magnetic configurations discussed earlier, it is also possible that the axes of the bubbles can become pinned so their tilting angles do not correspond to the specimen tilt.

Thus we conclude that there is no need to invoke a new magnetic state to explain the appearance of the images previously identified as biskyrmions. Such images, whether Yin‐Yang in appearance like those in Figure 2c of ref. 18 and Figures 2a and 3 of ref. 28 or half‐black half‐white as seen in refs. 18, 19, 20, 40, 41 can be explained as conventional type‐II bubbles with topological charge 0, viewed at an angle to their symmetry axes. Similar conclusions were reached in a recent preprint^43^ although the authors do not make a direct comparison between simulated and experimental images.

## Conflict of Interest

The authors declare no conflict of interest.

## Supporting information

Supplementary

Supplementary
