# Supplementary material for: Do Images of Biskyrmions Show Type‐II Bubbles?
Source: Adv Mater. 2019 Mar 7;31(16):1806598. doi: 10.1002/adma.201806598 (PMC9285551; doi:10.1002/adma.201806598)
Supplement: Supplementary file 1 — Supplementary [file ADMA-31-1806598-s002.pdf]

Copyright WILEY-VCH Verlag GmbH & Co. KGaA, 69469 Weinheim, Germany, 2019.

ADVANCED MATERIALS

Supporting Information

for *Adv. Mater.*, DOI: 10.1002/adma.201806598

Do Images of Biskyrmions Show Type-II Bubbles?

James C. Loudon, Alison C. Twitchett-Harrison, David Cortés-Ortuño, Max T. Birch, Luke A. Turnbull, Aleš Štefanc#ic#, Feodor Y. Ogrin, Erick O. Burgos-Parra, Nicholas Bukin, Angus Laurenson, Horia Popescu, Marijan Beg, Ondrej Hovorka, Hans Fangohr, Paul A. Midgley, Geetha Balakrishnan, and Peter D. Hatton*

Do Images of Biskyrmions Show Type-II Bubbles? – Supporting Information.

J. C. Loudon,[1,*] A. C. Twitchett-Harrison,[1] D. Cortés-Ortuño,[2] M. T. Birch,[3] L. A. Turnbull,[3] A. Štefančič,[4] F. Y. Ogrin,[5] E. Burgos-Parra,[5] N. Bukin,[5] A. Laurenson,[5] H. Popescu,[6] M. Beg,[2,7] O. Hovorka,[2] H. Fangohr,[7,2] P. A. Midgley,[1] G. Balakrishnan,[4] and P. D. Hatton[3]

[1]*Department of Materials Science and Metallurgy, University of Cambridge, 27 Charles Babbage Road, Cambridge, CB3 0FS, United Kingdom.*
[2]*Faculty of Engineering and Physical Sciences, University of Southampton, Southampton, SO17 1BJ, United Kingdom.*
[3]*Department of Physics, University of Durham, Durham, DH1 3LE, United Kingdom.*
[4]*Department of Physics, University of Warwick, Coventry, CV4 7AL, United Kingdom.*
[5]*School of Physics and Astronomy, University of Exeter, Exeter, EX4 4QL, United Kingdom.*
[6]*Synchrotron SOLEIL, Saint Aubin, BP 48, 91192 Gif-sur-Yvette, France.*
[7]*European XFEL GmbH, Holzkoppel 4, 22869 Schenefeld, Germany.*
(Dated: January 28, 2019)

S1. SAMPLE SYNTHESIS AND CHARACTERIZATION

Polycrystalline samples of MnNiGa with a target composition of $(Mn_{0.5}Ni_{0.5})_{0.65}Ga_{0.35}$ were prepared by arc-melting pellets of stoichiometric amounts of manganese (99.99%, Sigma-Adrich) and nickel (99.9%, Alfa Aesar) powders and gallium shots (99.99%, Alfa Aesar) in an arc furnace under a high-purity (5N) argon atmosphere. A 2 mol% excess of Mn was added to compensate for losses during the arc-melting procedure. The sample buttons were melted and flipped several times to ensure homogeneity. As-prepared buttons were annealed in vacuum at 800°C for 1 week to stabilise the structure by removing strains and potential disorder and to enlarge the grains before being quenched in water to retain the high temperature structure.

The phase-purity was determined by x-ray diffraction using a Panalytical X-Pert Pro powder x-ray diffractometer operating in Bragg-Brentano geometry. This showed that the crystal was hexagonal $P6_3/mmc$ (space group 194) with lattice parameters $a = b = 4.15125(3)$ Å and $c = 5.32774(8)$ Å which is in good agreement with the structure reported in refs. 1 and 2. According to both references, Ga atoms occupy the $2c$ sites with Mn preferentially occupying the $2a$ and Ni preferentially occupying the $2d$ sites (see Figure S1).

The composition was determined to be $Mn_{0.325(1)}Ni_{0.324(1)}Ga_{0.350(1)}$ by energy dispersive x-ray spectroscopy conducted using a Zeiss SUPRA 55-VP scanning electron microscope equipped with large area SDD EDX detector. (The number in brackets indicates the error in the last decimal place.)

A Quantum Design Magnetic Property Measurement System, MPMS-5S, superconducting quantum interference device (SQUID) magnetometer was used to investigate the magnetic properties of the polycrystalline samples. The Curie temperature was $T_C = 340 \pm 5$ K and the saturation magnetization at 5 K under an applied field of 5 T was 1.66 ± 0.02 μ_B per formula unit, both in good agreement with previously reported values [2].

S2. SAMPLE PREPARATION

MnNiGa samples were prepared for electron microscopy and x-ray holography using an FEI NanoLab-600 Helios Dual-Beam focused ion beam (FIB) microscope equipped with an Omniprobe-200 micromanipulator. Samples of approximate size 10 × 5 μm were cut from a single grain using gallium ion milling, lifted out with the micromanipulator and mounted on Omniprobe grids. As the orientation of the grain was unknown, two initial samples were cut parallel to the grain boundaries of the material and their orientation found *ex-situ* using the transmission electron microscope. The first sample was close to the [100] zone axis and showed no magnetic contrast, the second was close to the [011] and showed only striped magnetic contrast. From these, the tilting angles required to cut a sample in the [001] zone axis were calculated and the final samples were cut within 7° of the [001] zone axis. Two samples were cut from the same grain and thinned to about 200 nm. One sample

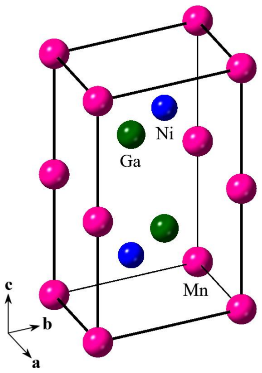

FIG. S1. Crystal structure of $Mn_{1/3}Ni_{1/3}Ga_{1/3}$.

* j.c.loudon@gmail.com

was used for x-ray holography and the other for transmission electron microscopy. Both striped magnetic domains and bubbles were observed in this orientation in agreement with the findings of Wang *et al.* [2].

S3. X-RAY HOLOGRAPHY

MnNiGa samples were prepared as described above and mounted on 3 μm diameter circular apertures patterned on Si_3Ni_4 membranes which had been coated with a 600 nm thick gold mask. Reference slits were cut alongside these holes, 6000 × 10 nm in size. The membranes were mounted in a vacuum chamber, containing an Halbach array, capable of applying a magnetic field of up to 0.5 T in any direction. A thermal resistor in contact with the sample was used to control the temperature.

X-ray holography was undertaken on the COMET instrument at the SEXTANTS beamline at SOLEIL, using holography with extended reference by autocorrelation linear differential operator (HERALDO) [3]. In this technique, a reference wave is produced using an extended slit instead of the more common pinhole aperture, leading to an improved signal-to-noise ratio and spatial resolution [4]. Magnetic contrast was generated by exploiting x-ray magnetic circular dichroism (XMCD) at the L_3 absorption edge of Mn at 637.5 eV. Far field diffraction patterns formed via the interference of the sample and reference aperture were recorded using a 2048 × 2048 pixel charge-coupled device (CCD). By taking the difference between patterns recorded with opposite x-ray helicity then applying a linear differential filter and a Fourier transform, reconstructed images of the local magnetization in the samples were obtained. The x-ray polarization was controlled with an HU44 APPLE II undulator [5].

S4. ELECTRON MICROSCOPY

Electron microscopy was undertaken using an FEI Titan^3 80-300 transmission electron microscope operated at an acceleration voltage of 300 kV and equipped with a high-brightness XFEG electron gun which increased the electron count in our images by a factor of about ten times compared with microscopes equipped with conventional field-emission guns. This allowed us to acquire meaningful images at low defocus despite the low contrast.

In normal operation, the electromagnetic objective lens applies a 2 T field to the specimen which would force it into the saturated state. Images were instead acquired in low-magnification mode where the image is formed using the diffraction lens and the objective lens was weakly excited to apply a small magnetic field parallel to the electron beam. The applied field corresponding a given objective lens current had been calibrated to within 1 mT using an FEI Hall probe holder.

Images were filtered using a Gatan 865 Tridiem energy-filter so that only electrons which had lost between 0 and 10 eV on passing through the specimen contributed to the image and an aperture subtending a half-angle of 0.14 mrad was used to ensure that only the 000 beam and those associated with magnetic scattering contributed to the image. Images were recorded on a 2048 × 2048 pixel charge-coupled device (CCD).

The defocus and magnification were calibrated by acquiring images with the same lens settings from Agar Scientific's S106 calibration specimen which consists of lines spaced by 463 nm ruled on an amorphous film. The defocus was found by taking digital Fourier transforms of these images and measuring the radii of the dark rings that result from the contrast transfer function of the lens using the method described in ref. 6 (ch. 28).

The specimen thickness was measured by taking in-focus images of the same area with and without energy filtering. Dividing the unfiltered image by the filtered image and taking the natural logarithm gives the thickness as a multiple of the inelastic mean free path of the electrons – a 't-over-lambda' map [6] (ch. 39.5). The absolute specimen thickness was measured at 15 positions using the two-beam convergent-beam electron diffraction technique described in ref. [6] (ch. 21.2) and the inelastic mean free path calibrated from this, giving a map of the absolute thickness across the whole specimen to within 10 nm accuracy.

S5. MICROMAGNETIC SIMULATIONS

We performed micromagnetic simulations using the Object Oriented Micro-Magnetic Framework (OOMMF) code [7, 8]. From the domain wall width of $\delta \approx 47\,\mathrm{nm}$ we estimate a ratio between the exchange constant and uniaxial anisotropy of the system as $A/K_\mathrm{u} = 2.3 \times 10^{-16}\,\mathrm{m}^2$. In a recent study, Zuo et al. [9] reported a magnitude for the saturation magnetization of $\mu_0 M_\mathrm{s} = 0.547\,\mathrm{T}$. In order to specify a exchange constant magnitude A, we did a parameter sweep starting with a hexagonal array of bubbles in $1\,\mu\mathrm{m} \times 1\,\mu\mathrm{m} \times 200\,\mathrm{nm}$ samples and varying M_s, A and the applied field. From the results, we observed the stabilization of bubbles around fields of 0.2 T for $\mu_0 M_\mathrm{s} = 0.550\,\mathrm{T}$ and for A between 10 and $20\,\mathrm{pJm}^{-1}$. By decreasing M_s, bubbles are observed when decreasing the exchange constant but at very weak fields. In addition, setting $\mu_0 M_\mathrm{s} \leq 0.550\,\mathrm{T}$, it was not possible to stabilize an isolated bubble at fields close to 0.2 T. From the simulations, a larger M_s magnitude allows the stabilization of both bubble arrays and isolated bubbles around 0.2 T. Thus, we chose an exchange field of of $20\,\mathrm{pJm}^{-1}$, and a saturation magnetization of $\mu_0 M_\mathrm{s} = 0.648\,\mathrm{T}$. Any magnitude of A and M_s around these values should also be valid for the stabilization of the bubbles. An optimal choice of micromagnetic parameters is beyond the scope of this work, since the observed experimental structures are in agreement with our simulations. With our choice

of parameters, we estimate a quality factor Q of about 0.52.

Using the magnetic parameters discussed previously, we analyzed the possibility of generating bi-skyrmion configurations through simulated hysteresis-like processes. We defined cuboid samples of 1 μm × 1 μm × 100 nm size considering exchange, dipolar and uniaxial anisotropy interactions. We discretized the system using cells of 4 nm × 4 nm × 4 nm volume, whose dimensions are well below the exchange length constant of the material $l_{\mathrm{ex}} = \sqrt{2A(\mu_0 M_{\mathrm{s}}^2)^{-1}}$ of 10.94 nm magnitude. The systems are initialized at zero field with randomly oriented spins. After energy minimization we found spiral configurations with varying chirality, as shown in Figure S2 and Video S1.

Across the thickness of the sample the helicities of the domain walls change from a Bloch-like profile at the center of the sample to a Néel-like profile towards the surfaces like that shown in Figure 4(a) (main text). Interestingly, we notice the presence of adjacent domain walls with the same chirality. Increasing the field up to 0.2 T, we observe the formation of the elongated spiral domains. Among them we notice elongated spiral domains surrounded by domain walls of equal chirality. This occurs by the presence of Bloch lines at the extremes of the longest domain dimension. Furthermore, we observe the emergence of different types of bubbles which coexist with the spiral domains: ordinary bubbles, which are of Néel type at the top and bottom surfaces of the sample and Bloch type at the sample center (across the thickness). We also find type-II bubbles of ellipsoidal shape, which are characterized by two Bloch lines at the extremes of the bubble long axis, that propagate through the sample thickness. Above fields of 0.2 T, only isolated bubbles remain in the system. Type-II bubbles are observed for fields below 0.26 T. From these simulations we did not directly find a magnetic ordering resembling a bi-skyrmion, however we noticed that the type-II bubbles have a profile that, averaged through the sample thickness, can generate a bi-skyrmionic state. Hence we focused our attention to isolated bubbles.

Simulations of isolated type-II bubbles were performed in samples of L μm × L μm × 200 nm volume, with L varying from 600 nm up to 1.2 μm. As a initial state we specified a Bloch-like bubble propagating through the thickness of the sample, with the upper and lower half of the bubble (along the y-direction) with opposite chiralities. After energy minimization, at applied fields below 0.9 T we find elongated domains surrounded by domain walls of the same chirality. At the surfaces of the sample the helicity of the domain wall defining the stripe domains vary as in the hysteresis-like simulations. At sufficiently strong applied fields we find isolated type-II bubbles of ellipsoidal shape with the same varying helicity profile of the stripe domains across the thickness. With increasing fields the bubble profile becomes more spherical and decreases in size, as shown in Figure S3. This figure also indicates that the stability of these bubbles is affected by varying the side lengths of the sample, thus isolated type-II bubbles are not observed in infinite systems. Moreover, they are only observed up to a field of 0.26 T, which might change by varying the saturation magnetization or the exchange constant values. In contrast, in Figure S2 we observe that type-I bubble arrays survive in a larger field range. When decreasing the system thickness, we saw from simulations that type-II bubbles are stable in a more restricted range of applied field magnitudes.

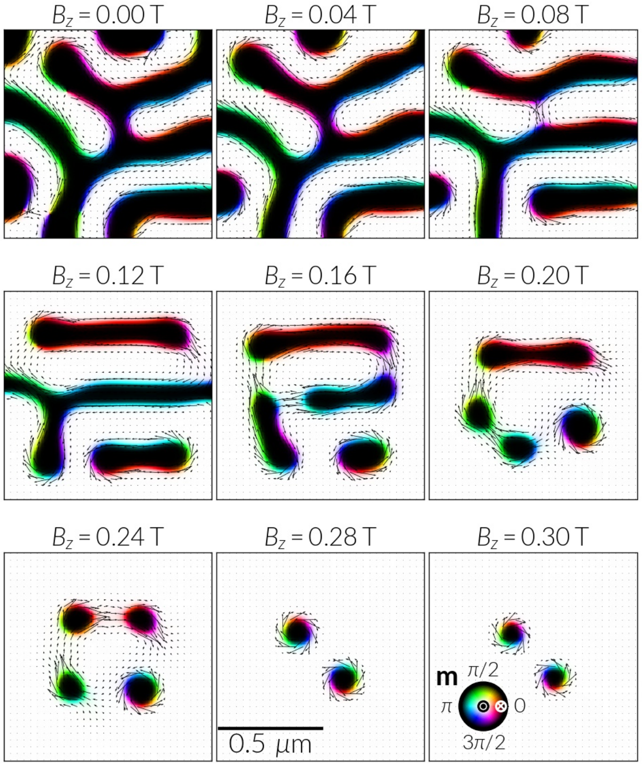

FIG. S2. Micromagnetic simulation of a field sweep process on a MnNiGa sample of 1 μm size and 100 nm thickness. The initial state was generated from randomly oriented spins. The hysteresis-like process was simulated using steps of 20 mT. Snapshots are taken from a slice at the center of the sample, perpendicular to the z-direction. Video S1 shows the same process as a movie.

Figure S4 shows the effect on a simulated magnetic bubble of changing the specimen thickness. It can be seen that the bubbles in thinner specimens are smaller and more elliptical with their major axis following a line connecting the internal domain walls. All the bubbles share the feature that the magnetization points radially at the top and the bottom layers and the magnetization twists throughout the specimen thickness, showing the classic type-II configuration only in the middle layer.

All the simulation data can be reproduced from the repository given in Ref. [10].

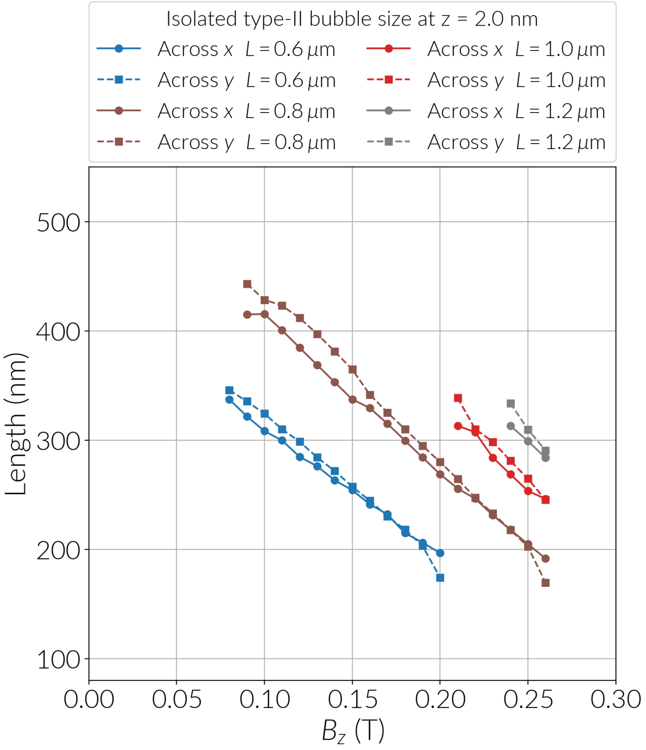

FIG. S3. Size of an isolated Type-II bubble in cuboid samples of varying width and a thickness of 200 nm. The bubble size is measured as the length between spins with $m_z = 0$ in a line across the bubble center. The plot shows both when the line is in the x and y direction, thus indicating an ellipsoidal shape of the bubble when the distances have different length.

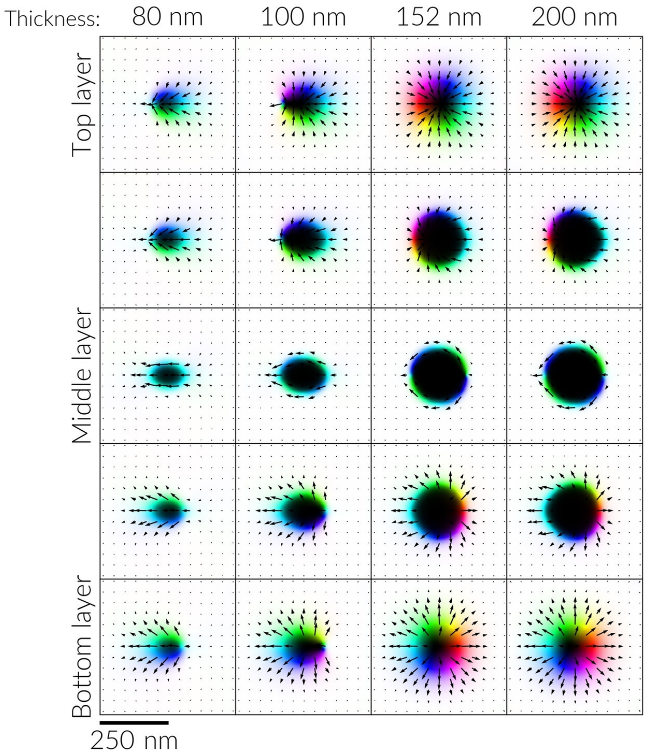

FIG. S4. Simulated magnetic bubbles for different specimen thicknesses for an out-of-plane applied field of 240 mT. Each column shows the magnetization of a single bubble with the specimen thickness given at the top. Each panel in the column shows the magnetization at equally spaced layers parallel to the specimen surfaces from the top to the bottom of the specimen. The direction and strength of the magnetization is indicated by arrows and colors using the same color scheme as figure S2. Note that for specimens thinner than 80 nm, bubbles could not be stabilized and the saturated state was obtained.

S6. MAGNETIC BUBBLES FROM DIFFERENT SPECIMEN THICKNESSES

Figure S5(a) shows an image from a defocus series with stripe domains and magnetic bubbles coexisting. The sample was cut to two different thicknesses with a thickness step running nearly vertically down the middle. The thickness was measured as described in section S4. The thinner region on the left appears lighter and has a gradual increase in thickness from 110 nm at the top of the image to 180 nm at the bottom. The thicker region on the right is 200 nm thick at the top and increases to 230 nm at the bottom. The straight lines parallel to the thickness step in the thinner region are slight thickness undulations of about 1 nm caused by the focused ion beam milling used to prepare the specimen.

The bubbles have a mean spacing of 220 nm in the thinner region and 310 nm in the thicker region, both with a standard deviation of 25 nm. They have a mean diameter of 72 nm in the thinner region with a standard deviation of 13 nm and a mean diameter of 117 nm with a standard deviation of 6 nm in the thicker region. This follows the trend shown by the simulations that the bubbles in thicker specimens tend to be larger.

The bubbles in both regions exhibit a similar Yin-Yang appearance although the black-white contrast of the bubbles in the thinner region is rotated with respect to those in the thick region. Simulations show that this rotation reflects the positions of the internal domain walls. A closer inspection shows that within the same region, neighboring bubbles show small rotations with respect to one another.

The transport of intensity equation was used to reconstruct the projected B-field. The B-field from the bubble in the thinner region outlined in blue is shown in (b) and the B-field from a bubble in the thicker region outlined in red is shown in (c). The bubble from the thinner region is from a thickness of 180 nm and has a diameter of 87 nm while the bubble from the thicker region is from a thickness of 220 nm and has a diameter of 117 nm. It can be seen that the pattern of B-field lines is very similar in both cases and closely resembles the B-field in figure 3 of the main paper. This indicates that although the thickness of the specimen and the size and spacing of the bubbles varies, all the bubbles shown in the image are

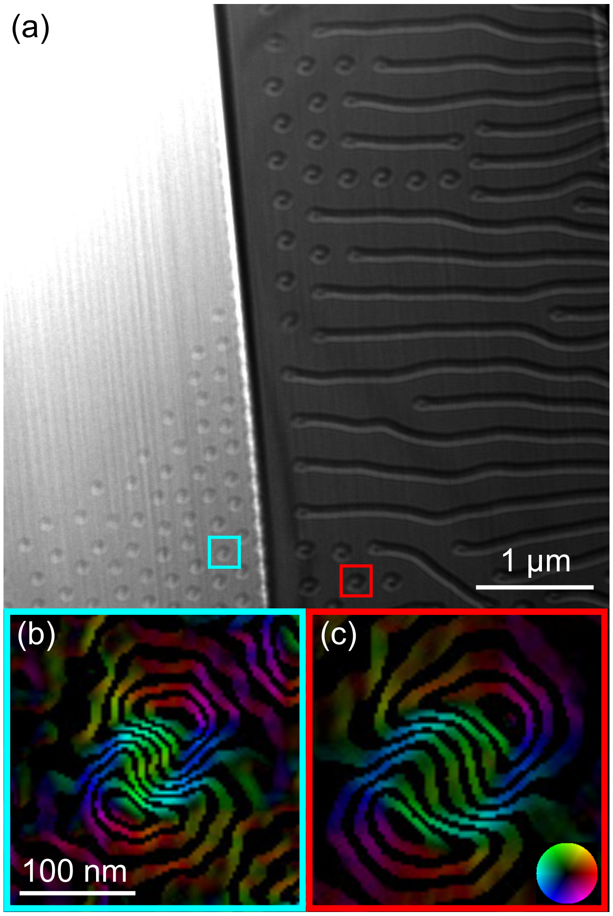

FIG. S5. (a) Electron micrograph showing magnetic bubbles and stripe domains in MnNiGa in regions of specimen with different thickness at room temperature in an out-of-plane applied field of 233 mT. The image has a defocus $\Delta f = -1.410$ mm and is a member of a defocus series taken under conditions identical to figure 3 of the main paper. (b) Projected B-field for a bubble from the thinner region in (a) indicated by the blue square reconstructed from the transport of intensity equation. (c) Projected B-field for a bubble from the thicker region in (a) indicated by the red square and reconstructed in the same way. The directions of the B-field are shown by the color wheel and the B-field lines correspond to the cosine of 100 times the phase of the exit wavefunction of the electron beam.

type-II.

S7. SIMULATION OF ELECTRON MICROGRAPHS

A. Simulation of Defocused Images

Once a model for the magnetization in a specimen has been obtained, electron micrographs can be simulated as follows. The first step is to find the magnetic vector potential. This can be done by splitting the specimen into an infinite number of magnetic dipoles and summing the vector potentials from each. Thus the vector potential $\mathbf{A}(\mathbf{r})$ can be related to the magnetization $\mathbf{M}(\mathbf{r})$ via:

$$\mathbf{A}(\mathbf{r}) = \frac{\mu_0}{4\pi} \int \mathbf{M}(\mathbf{R}) \times \left(\frac{\mathbf{r} - \mathbf{R}}{|\mathbf{r} - \mathbf{R}|^3} \right) \mathrm{d}^3\mathbf{R}. \quad (1)$$

where $\mathbf{r}$ and $\mathbf{R}$ are 3 dimensional position vectors and μ_0 is the permeability of free space. As this is a convolution, it is treated most simply as a multiplication in Fourier space [11]. Taking the 3-dimensional Fourier transform gives:

$$\widetilde{\mathbf{A}}(\mathbf{k}) = -\frac{i\mu_0}{2\pi} \frac{\widetilde{\mathbf{M}}(\mathbf{k}) \times \mathbf{k}}{k^2}, \quad (2)$$

Taking Cartesian coordinates in which the initial electron beam direction is parallel to z, the phase shift the electron beam has experienced when it exits the specimen is related to the vector potential via the Aharanov-Bohm formula [12]:

$$\phi(x, y) = -\frac{2\pi e}{h} \int_{-\infty}^{\infty} A_z(x, y, z) \mathrm{d}z. \quad (3)$$

In Fourier space, this becomes:

$$\widetilde{\phi}(k_x, k_y) = -\frac{2\pi e}{h} \widetilde{A}_z(k_x, k_y, 0). \quad (4)$$

and so the phase can be calculated directly from the magnetization using the formula given by Beleggia and Zhu [11]:

$$\widetilde{\phi}(k_x, k_y) = \frac{ied\mu_0}{h} \frac{\left[\widetilde{\mathbf{M}}_\perp(k_x, k_y) \times \mathbf{k}_\perp\right]_z}{k_\perp^2}, \quad (5)$$

where d is the specimen thickness, $\mathbf{k}_\perp \equiv (k_x, k_y, 0)$ and $\widetilde{\mathbf{M}}_\perp(k_x, k_y)$ is the two-dimensional Fourier transform of the magnetization averaged along z through the thickness of the sample.

The in-focus wavefunction is then $\psi_0 = \exp[i\phi]$ and it can be seen immediately that an in-focus image of a purely magnetic specimen will be featureless as $I_0 = |\psi_0|^2 = 1$. One way to obtain an image showing magnetic contrast is to take images out of focus. This technique is called defocused or Fresnel imaging and is often referred to as Lorentz imaging or LTEM although the latter term should encompass all the magnetic imaging techniques available with an electron microscope rather than this specific method. The wavefunction for a defocused image $\psi_{\Delta f}$ can be calculated using the Fresnel-Kirchoff integral [13, 14] which describes the propagation of waves through free space and can be written as a convolution:

$$\psi_{\Delta f} = \psi_0 * \left(\frac{e^{i\pi r^2/\lambda\Delta f}}{\lambda|\Delta f|} \right) \quad (6)$$

where $r^2 = x^2 + y^2$ and $*$ indicates a convolution. The calculation of the defocused image is usually performed in Fourier space as the convolution becomes a multiplication. Thus

$$\widetilde{\psi}_{\Delta f} = \widetilde{\psi}_0 e^{-i\pi\lambda\Delta f k^2} \tag{7}$$

The defocused image is then $I_{\Delta f} = |\psi_{\Delta f}|^2$.

B. Projected B-field

It is often of interest to calculate the projected B-field, $\mathbf{B}_\perp$. This is the component of the magnetic flux density normal to the electron beam averaged along the trajectory of an unscattered electron beam. If Stokes' theorem is applied to the Aharanov-Bohm equation (Eqn. 3) and the magnetic flux density substituted for the curl of the vector potential, the resulting integral can be inverted to give the projected B-field as:

$$\mathbf{B}_\perp(x,y) = \frac{h}{2\pi e d}\begin{pmatrix} -\partial/\partial y \\ \partial/\partial x \end{pmatrix}\phi \tag{8}$$

C. Analytic Model for a Type-I Magnetic Bubble

Thus, if we know the magnetization, the method described above allows us to simulate images that would be acquired using transmission electron microscopy. Here we describe the analytic model for the magnetization of a magnetic bubble used for comparison with experimental images. In this model, it is assumed the magnetization does not to vary through the specimen thickness and so represents the magnetization averaged through the thickness along the electron beam direction. Using a Cartesian coordinate system in which the axis of the bubble is parallel to the z-axis, a standard model [11, 15] for a circular magnetic domain wall has in-plane magnetization:

$$\mathbf{M}_{xy} = M_s \operatorname{sech}\left(\frac{r-R}{\delta/\pi}\right)\begin{pmatrix} -\sin\theta \\ \cos\theta \end{pmatrix} \tag{9}$$

and z-component

$$M_z = M_s \tanh\left(\frac{r-R}{\delta/\pi}\right) \tag{10}$$

where r is the distance from the bubble's axis and θ is the azimuthal angle. The constants are: M_s the saturation magnetization, R the radius of the magnetic bubble and δ the domain wall width. The domain wall width is given by $\delta = \pi\sqrt{A/K}$ with A being the exchange energy constant and K the uniaxial magnetocrystalline anisotropy.

If the magnetization direction does not follow the domain wall but deviates from it by a constant angle θ_d, the z-component remains the same whereas the in-plane component becomes:

$$\mathbf{M}_{xy} = M_s \operatorname{sech}\left(\frac{r-R}{\delta/\pi}\right)\begin{pmatrix} -\sin(\theta+\theta_d) \\ \cos(\theta+\theta_d) \end{pmatrix} \tag{11}$$

A purely Bloch bubble is produced when $\theta_d = 0$ and a Néel bubble is produced when $\theta_d = 90°$.

D. Field Lines for a Magnetic Bubble

It is beneficial to calculate the shape of the field lines for the magnetization of a magnetic bubble as an internal domain wall will likely follow a field line of $\mathbf{M}$ to avoid generating magnetic poles. Coey [16] (p 28 eqn. 2.11) defines a field line as a line which solves

$$\frac{dr}{r d\theta} = \frac{M_r}{M_\theta} \tag{12}$$

Expressing Eqn. 11 in polar coordinates gives

$$\mathbf{M}_{xy} = M_s \operatorname{sech}\left(\frac{r-R}{\delta/\pi}\right)\left[-\sin\theta_d\,\widehat{\mathbf{r}} + \cos\theta_d\,\widehat{\boldsymbol{\theta}}\right] \tag{13}$$

So the magnetic field lines are the solution to

$$\frac{dr}{r d\theta} = \frac{M_r}{M_\theta} = -\tan\theta_d \tag{14}$$

Solving this differential equation gives the equation of a logarithmic spiral [17] so that a field line going through a point (R, θ_i) has equation:

$$\theta = \theta_i + \cot(\theta_d)\ln(R/r) \tag{15}$$

E. Model for a Modified Type-II Magnetic Bubble

A type-II bubble can be modeled by adding a reversing function θ_{rev} to Eqn. 11:

$$\mathbf{M}_{xy} = M_s \operatorname{sech}\left(\frac{r-R}{\delta/\pi}\right)\begin{pmatrix} -\sin(\theta+\theta_d+\theta_{\text{rev}}) \\ \cos(\theta+\theta_d+\theta_{\text{rev}}) \end{pmatrix} \tag{16}$$

The reversing function adds π to the angle the magnetization makes with the radius between the two internal domain walls which occur at angles θ_1, θ_2. If the internal domain walls run radially, the reversing function can be written as:

$$\theta_{\text{rev}} = \pm\operatorname{atan}\left(\sinh\left(\frac{\pi r}{\delta_i}(\theta-\theta_1)\right)\right) \pm\operatorname{atan}\left(\sinh\left(\frac{\pi r}{\delta_i}(\theta-\theta_2)\right)\right) \tag{17}$$

where the arctangent of the hyperbolic sine is the function describing the magnetization direction in a domain wall given by Coey [16] and δ_i is the internal domain wall width.

The magnetization of each of the internal domain walls can point towards or away from the center of the bubble. In combination with this, the magnetization of the bubble can circulate in one of two directions leading to eight possible domain wall arrangements. Each of these can be selected by reversing the sign of M_s in Eqn. 16 in combination with choosing the sign of the arctangent functions in Eqn. 17.

When $\theta_d = 0$, this model represents a conventional type-II bubble. When θ_d is non-zero, it may be that the internal domain wall follows a field line of magnetization to avoid generating magnetic poles and thereby minimize stray field energy. The equation for the field lines (Eqn. 15) can be used so that if the walls go through the points (R, θ_1) and (R, θ_2), the reversing function becomes:

$$\theta_{\text{rev}} = \pm\,\text{atan}\left(\sinh\left(\frac{\pi r}{\delta_i}\left(\theta - \theta_1 - \cot(\theta_d)\ln(R/r)\right)\right)\right) \pm\,\text{atan}\left(\sinh\left(\frac{\pi r}{\delta_i}\left(\theta - \theta_2 - \cot(\theta_d)\ln(R/r)\right)\right)\right) \quad (18)$$

F. Modified Type-II Magnetic Bubble with an Outer Domain Wall

A comparison with experimental images indicated that the bubble had an outer domain wall. To accommodate this, we allowed the deviation angle θ_d to vary between two limits: θ_i near the center and θ_o far from the center with a domain wall at radius R_o and width δ_o. Thus we have the following model for a magnetic bubble which is the analytic model used in the main text for comparison with the experimental images:

$$\mathbf{M}_{xy} = M_s\,\text{sech}\left(\frac{r-R}{\delta/\pi}\right)\begin{pmatrix}-\sin(\theta+\theta_d+\theta_{\text{rev}})\\ \cos(\theta+\theta_d+\theta_{\text{rev}})\end{pmatrix} \quad (19)$$

where

$$\theta_{\text{rev}} = \pm\,\text{atan}\left(\sinh\left(\frac{\pi r}{\delta_i}\left(\theta - \theta_1 - \cot(\theta_d)\ln(R/r)\right)\right)\right) \pm\,\text{atan}\left(\sinh\left(\frac{\pi r}{\delta_i}\left(\theta - \theta_2 - \cot(\theta_d)\ln(R/r)\right)\right)\right) \quad (20)$$

and

$$\theta_d = \theta_i + \left(\text{atan}\left(\sinh\left(\frac{\pi}{\delta_o}(r - R_o)\right)\right) + \frac{\pi}{2}\right) \times \left(\frac{\theta_o - \theta_i}{\pi}\right) \quad (21)$$

This model was compared with the experimental images of 19 bubbles as described in the main text, yielding the parameters given in Table S1.

TABLE S1. Parameters Used in the Analytic Model of a Magnetic Bubble.

Saturation magnetization	$\mu_0 M_s$	0.0551 ± 0.0006 T
Domain wall width	δ	47 ± 1 nm
Internal domain wall width	δ_i	55 ± 1 nm
Outer domain wall width	δ_o	31 ± 1 nm
Major axis of bubble	R	56.6 ± 0.7 nm
Major axis of outer domain wall	R_o	90 ± 1 nm
Internal domain wall 1	θ_1	$32.8 \pm 1.6°$
Internal domain wall 2	θ_2	$214.9 \pm 0.9°$
Inner deviation angle	θ_i	$131 \pm 1°$
Outer deviation angle	θ_o	$32 \pm 2°$
Major-axis tilt	θ_t	$-11.4 \pm 0.8°$
Major to minor axis ratio		1.25 ± 0.02
χ^2		7.3

[1] H. Shiraishi, H. Niida, Y. Iguchi, S. Mitsudo, M. Motokawa, K. Ohayama, H. Miki, H. Onodera, T. Hori, K. Kanematsu. *J. Magn. Magn. Mater.* **1999**, *196* 660.

[2] W. Wang, Y. Zhang, G. Xu, L. Peng, B. Ding, Y. Wang, Z. Hou, X. Zhang, X. Li, E. Liu, S. Wang, J. Cai, F. Wang, J. Li, F. Hu, G. Wu, B. Shen, X.-X. Zhang. *Adv. Mater.* **2016**, *28* 6887.

[3] T. A. Duckworth, F. Ogrin, S. S. Dhesi, S. Langridge, A. Whiteside, T. Moore, G. Beutier, G. van der Laan. *Optics Express* **2011**, *19* 16223.

[4] V. Tenner, K. S. E. Eikema, S. Witte. *Optics Express* **2014**, *22* 25397.

[5] M. Sacchi, N. Jaouen, H. Popescu, R. Gaudemer, J. M. Tonnerre, S. G.Chiuzbaian, C. F. Hauge, A. Delmotte, J. M. Dubuisson, B. Lagarde, F. Polack. *Journal of Physics: Conference Series* **2013**, *425*, 7 072018.

[6] D. B. Williams, C. B. Carter. *Transmission Electron Microscopy.* Springer, New York, USA, 2nd edition, **2009**.

[7] M. J. Donahue, D. G. Porter. Oommf users guide, version 1.0. Technical report, National Institute of Standards and Technology, Gaithersburg, MD, **1999**.

[8] M. Beg, R. A. Pepper, H. Fangohr. *AIP Advances* **2017**, *7*, 5 056025.

[9] S. L. Zuo, Y. Zhang, L. C. Peng, X. Zhao, R. Li, H. Li, J. F. Xiong, M. He, T. Y. Zhao, J. R. Sun, F. X. Hu, B. G. Shen. *Nanoscale* **2018**, *10* 2260.

[10] D. Cortés-Ortuño, J. C. Loudon, M. Beg, O. Hovorka, H. Fangohr. Supplementary data for: Do Images of Biskyrmions Show Type-II Bubbles? Zenodo doi:10.5281/zenodo.1420447. Github:https://github.com/davidcortesortuno/paper_biskyrmions_bubbles, **2019**.
[11] M. Beleggia, Y. Zhu. *Phil. Mag.* **2003**, *83* 1045.
[12] Y. Aharanov, D. Bohm. *Phys. Rev.* **1959**, *115* 485.
[13] E. Hecht. *Optics, 2nd edtn.* Addison-Wesley, Massachusetts, USA, **1974**.
[14] M. Beleggia, G. Pozzi. *Phys. Rev. B* **2001**, *63* 054507.
[15] M. Mansuripur. *The Physical Principles of Magneto-Optical Recording*. Cambridge University Press, Cambridge, UK, **1995**.
[16] J. M. D. Coey. *Magnetism and Magnetic Materials*. Cambridge University Press, Cambridge, UK, **2010**.
[17] E. H. Lockwood. *A Book of Curves*. Cambridge University Press, Cambridge, UK, **1963**.
